# Supplementary material for: Real Life Clinical Impact of Antimicrobial Stewardship Actions on the Blood Culture Workflow from a Microbiology Laboratory
Source: Antibiotics (Basel). 2021 Dec 9;10(12):1511. doi: 10.3390/antibiotics10121511 (PMC8698396; doi:10.3390/antibiotics10121511)
Supplement: Supplementary file 1 [file antibiotics-10-01511-s001.zip › antibiotics-1454871-supplementary.pdf]

antibiotics

MDPI

**Supplementary Materials:**

**Table S1.** LOS (median days [IQ1-IQ3]) in intervention and control group.

| GLOBAL | CONTROL GROUP | n | INTERVENTION GROUP | n | *p* |
|---|---|---|---|---|---|
| **General** | 8.00 [5.00-16.00] | 125 | 8.00 [5.00-16.00] | 188 | 0.756 |
| Category 1 | 4.00 [4.00-4.00] | 1 | 18.00 [9.00-35.00] | 12 | 0.109 |
| Category 2 | 8.00 [5.00-14.00] | 79 | 8.00 [5.00-14.00] | 120 | 0.532 |
| Category 3 | 28.00 [7.00-49.00] | 2 | 27.50 [5.00-14.00] | 10 | 0.776 |
| Category 4 | 10.50 [5.00-21.00] | 36 | 7.00 [5.00-15.00] | 34 | 0.266 |
| Category 5 | 7.00 [1.00-16.00] | 7 | 6.00 [1.00-7.50] | 12 | 0.831 |
| **Medical ward** | 9.00 [5.00-18.00] | 84 | 8.00 [5.00-15.00] | 121 | 0.246 |
| Category 1 | -- | 0 | 20.50 [8.00-30.00] | 6 | -- |
| Category 2 | 9.00 [6.00-16.00] | 51 | 8.00 [2.00-15.00] | 78 | 0.350 |
| Category 3 | 28.00 [7.00-49.00] | 2 | 8.00 [8.00-21.00] | 5 | 0.696 |
| Category 4 | 11.50 [5.00-24.00] | 26 | 8.00 [5.00-22.00] | 23 | 0.507 |
| Category 5 | 1.00 [1.00-7.00] | 5 | 4.00 [1.00-6.00] | 9 | 0.498 |
| **Surgical ward** | 9.00 [5.00-15.00] | 18 | 9.50 [5.00-15.00] | 38 | 0.923 |
| Category 1 | -- | 0 | 46.00 [15.00-51.00] | 3 | -- |
| Category 2 | 2.00 [2.00-2.00] | 12 | 10.00 [5.00-14.00] | 23 | 1.000 |
| Category 3 | -- | 0 | 79.50 [48.00-111.00] | 2 | -- |
| Category 4 | 7.00 [5.00-12.00] | 5 | 5.00 [4.00-8.50] | 8 | 0.232 |
| Category 5 | 11.00 [11.00-11.00] | 1 | 6.5 [6.00-7.00] | 2 | 0.221 |
| **Infectious diseases** | 5.00 [3.00-10.00] | 14 | 6.00 [5.00-9.00] | 17 | 0.318 |
| Category 1 | 4.00 [4.00-4.00] | 1 | 8.00 [6.00-10.00] | 2 | 0.221 |
| Category 2 | 5.00 [3.00-6.00] | 11 | 6.00 [5.00-8.00] | 14 | 0.281 |
| Category 3 | -- | 0 | -- | 0 | -- |
| Category 4 | 10.00 [10.00-10.00] | 2 | -- | 0 | -- |
| Category 5 | -- | 0 | -- | 0 | |
| **ICU** | 9.00 [7.00-16.00] | 9 | 23.00 [7.00-34.50] | 12 | 0.109 |
| Category 1 | -- | 0 | 21.00 [21.00-21.00] | 1 | -- |
| Category 2 | 9.00 [7.00-9.00] | 5 | 24.00 [22.00-34.00] | 5 | **0.026** |
| Category 3 | -- | 0 | 34.00 [4.00-84.00] | 3 | -- |
| Category 4 | 8.00 [7.00-22.00] | 3 | 5.00 [5.00-35.00] | 3 | 0.507 |
| Category 5 | 16.00 [16.00-16.00] | 1 | -- | 0 | -- |

**Table S2.** 7-days mortality (%) in control and intervention groups.

| GLOBAL | CONTROL GROUP | INTERVENTION GROUP | *p* |
|---|---|---|---|
| **General** | 4% (5/125) | 6% (12/188) | 0.362 |
| Category 1 | 0% (0/1) | 8% (1/12) | 0.764 |
| Category 2 | 4% (3/79) | 4% (5/120) | 0.897 |
| Category 3 | 0% (0/2) | 10% (1/10) | 0.640 |
| Category 4 | 6% (2/36) | 9% (3/34) | 0.596 |
| Category 5 | 0% (0/7) | 17% (2/12) | 0.253 |
| **Medical ward** | 5% (4/84) | 6% (7/121) | 0.749 |
| Category 1 | -- (0/0) | 17% (1/6) | -- |
| Category 2 | 4% (2/51) | 4% (3/78) | 0.983 |
| Category 3 | 0% (0/2) | 0% (0/5) | -- |

| | | | |
|---|---|---|---|
| Category 4 | 8% (2/26) | 4% (1/23) | 0.626 |
| Category 5 | 0% (0/5) | 22% (2/9) | 0.255 |
| **Infectious diseases** | 0% (0/14) | 0% (0/17) | -- |
| Category 1 | 0% (0/1) | 0% (0/2) | -- |
| Category 2 | 0% (0/11) | 0% (0/14) | -- |
| Category 3 | -- (0/0) | -- (0/0) | -- |
| Category 4 | 0% (0/2) | -- (0/0) | -- |
| Category 5 | -- (0/0) | 0% (0/1) | -- |
| **ICU** | 11% (1/9) | 25% (3/12) | 0.422 |
| Category 1 | -- (0/0) | 0% (0/1) | -- |
| Category 2 | 20% (1/5) | 0% (0/5) | 0.292 |
| Category 3 | -- (0/0) | 33% (1/3) | -- |
| Category 4 | 0% (0/3) | 67% (2/3) | 0.083 |
| Category 5 | 0% (0/1) | -- (0/0) | -- |

**Table S3.** 30-days mortality in control and intervention groups.

| GLOBAL | CONTROL GROUP | INTERVENTION GROUP | *p* |
|---|---|---|---|
| **General** | 5% (6/125) | 8% (15/188) | 0.271 |
| Category 1 | 0% (0/1) | 8% (1/12) | 0.764 |
| Category 2 | 5% (4/79) | 6% (7/120) | 0.816 |
| Category 3 | 0% (0/2) | 10% (1/10) | 0.640 |
| Category 4 | 6% (2/36) | 11% (4/34) | 0.354 |
| Category 5 | 0% (0/7) | 25% (2/12) | 0.253 |
| **Medical ward** | 6% (5/84) | 8% (10/121) | 0.532 |
| Category 1 | -- (0/0) | 17% (1/6) | -- |
| Category 2 | 6% (3/51) | 6% (5/78) | 0.903 |
| Category 3 | 0% (0/2) | 0% (0/5) | -- |
| Category 4 | 8% (2/26) | 9% (2/23) | 0.898 |
| Category 5 | 0% (0/5) | 22% (2/9) | 0.255 |
| **Surgical ward** | 0% (0/18) | 5% (2/38) | 0.322 |
| Category 1 | -- (0/0) | 0% (0/3) | -- |
| Category 2 | 0% (0/12) | 9% (2/23) | 0.293 |
| Category 3 | -- (0/0) | 0% (0/2) | -- |
| Category 4 | 0% (0/5) | 0% (0/8) | -- |
| Category 5 | 0% (0/1) | 0% (0/2) | -- |
| **Infectious diseases** | 0% (0/14) | 0% (0/17) | -- |
| Category 1 | 0% (0/1) | 0% (0/2) | -- |
| Category 2 | 0% (0/11) | 0% (0/14) | -- |
| Category 3 | -- (0/0) | -- (0/0) | -- |
| Category 4 | 0% (0/2) | -- (0/0) | -- |
| Category 5 | -- (0/0) | 0% (0/1) | -- |
| **ICU** | 11% (1/9) | 25% (3/12) | 0.422 |
| Category 1 | -- (0/0) | 0% (0/1) | -- |
| Category 2 | 20% (1/5) | 0% (0/5) | 0.292 |
| Category 3 | -- (0/0) | 33% (1/3) | -- |
| Category 4 | 0% (0/3) | 67% (2/3) | 0.083 |
| Category 5 | 0% (0/1) | -- (0/0) | -- |

**Table S4.** Percentage of readmissions for 30 days distributed by groups.

| GLOBAL | CONTROL GROUP | INTERVENTION GROUP | *p* |
|---|---|---|---|
| **General** | 10% (13/125) | 15% (29/188) | 0.201 |
| Category 1 | 0% (0/1) | 25% (3/12) | 0.569 |
| Category 2 | 14% (11/79) | 12% (14/120) | 0.638 |
| Category 3 | 0% (0/2) | 10% (1/10) | 0.640 |
| Category 4 | 6% (2/36) | 29% (10/34) | 0.008 |
| Category 5 | 0% (0/7) | 17% (1/12) | 0.433 |
| **Medical ward** | 12% (10/84) | 17% (20/121) | 0.357 |
| Category 1 | -- (0/0) | 33% (2/6) | -- |
| Category 2 | 16% (8/51) | 10% (8/78) | 0.360 |
| Category 3 | 0% (0/2) | 20% (1/5) | 0.495 |
| Category 4 | 8% (2/26) | 35% (8/23) | **0.019** |
| Category 5 | 0% (0/5) | 11% (1/9) | 0.439 |
| **Surgical ward** | 6% (1/18) | 16% (6/38) | 0.279 |
| Category 1 | -- (0/0) | 33% (1/3) | -- |
| Category 2 | 8% (1/12) | 13% (3/23) | 0.678 |
| Category 3 | -- (0/0) | 0% (0/2) | -- |
| Category 4 | 0% (0/5) | 25% (2/8) | 0.224 |
| Category 5 | 0% (0/1) | 0% (0/2) | -- |
| **Infectious diseases** | 0% (0/14) | 6% (1/17) | 0.356 |
| Category 1 | 0% (0/1) | 0% (0/2) | -- |
| Category 2 | 0% (0/11) | 7% (1/14) | 0.366 |
| Category 3 | -- (0/0) | -- (0/0) | -- |
| Category 4 | 0% (0/2) | -- (0/0) | -- |
| Category 5 | -- (0/0) | 0% (0/1) | -- |
| **ICU** | 2% (2/9) | 2% (2/12) | 0.748 |
| Category 1 | -- (0/0) | 0% (0/1) | -- |
| Category 2 | 40% (2/5) | 40% (2/5) | 1.000 |
| Category 3 | -- (0/0) | 0% (0/3) | -- |
| Category 4 | 0% (0/3) | 0% (0/3) | -- |
| Category 5 | 0% (0/1) | -- (0/0) | -- |
